# Evaluation of the expect respect support group program: A violence prevention strategy for youth exposed to violence

**DOI:** 10.1016/j.ypmed.2017.05.003

**Published:** 2017-05-11

**Authors:** Dennis E. Reidy, Kristin M. Holland, Kai Cortina, Barbara Ball, Barri Rosenbluth

**Affiliations:** a Division of Violence Prevention, Centers for Disease Control & Prevention, United States; b Department of Psychology, University of Michigan, United States; c SAFE Alliance, United States

**Keywords:** Teen dating violence, Prevention, Sexual violence, Reactive aggression, Proactive aggression, Dating violence prevention, Program evaluation

## Abstract

In the present study, we assess the effects of the Expect Respect Support Groups (ERSG) on frequency of teen dating violence (TDV) and general youth violence. ERSG is a school-based violence prevention program for youth who have been exposed to violence in their home, school, or community. Boys and girls (N=1,678, M_age_=14.3, S.D.=1.7, Range = 11–17) from 36 schools in Texas participated in this accelerated longitudinal (7-year trajectory) study beginning in 2011. Latent growth curve analyses were conducted using three waves of data from three cross-sectional cohorts of adolescents. Among boys, the number of ERSG sessions attended related to incremental declines in psychological TDV perpetration and victimization, physical TDV victimization, sexual TDV perpetration and victimization, reactive aggression, and proactive aggression. Girls attending ERSG demonstrated reductions in reactive and proactive aggression. The present findings suggest ERSG may be an effective cross-cutting strategy to reduce TDV and other forms of violence among high-risk boys and possibly girls. This information provides valuable understanding of TDV and youth violence in high-risk populations and may be useful in tailoring future prevention efforts to different groups of teens.

## 1. Introduction

Teen dating violence (TDV) is a serious public health problem with chronic deleterious consequences including psychiatric symptoms, substance use/abuse, poor educational attainment, etc. (Kann et al., 2014; Banyard and Cross, 2008; Ackard et al., 2007; Smith et al., 2003; White and Smith, 2004). Currently, few programs have shown success in reducing/preventing TDV. Taylor and colleagues (2015) examined the effectiveness of the universal ‘Shifting Boundaries,’ program which includes a classroom-based curriculum and a building-level intervention (i.e., school-based restraining orders, increased security in violence “hot spots,” and posters to increase awareness of sexual TDV). Findings indicated the building-level intervention reduced sexual TDV perpetration and victimization for boys and girls alike at six months post-intervention, but the curriculum component of the intervention had no effect. This distinction between environmental (i.e., building-level) and individual (i.e., curriculum) components is important because it suggests that the program reduced the opportunity, rather than the propensity, to perpetrate sexual violence. Thus, the prevention effects of this program may be less likely to generalize across settings and be sustained over time compared to strategies that are also effective at changing attributes of the individual.

Conversely, two existing curriculum-based programs have shown promise in preventing TDV. The universal school-based ‘Fourth-R’ curriculum demonstrated efficacy in reducing physical TDV perpetration among boys over 30-month follow-up (Wolfe et al., 2009). Foshee and colleagues (Foshee et al., 2005) confirmed the effectiveness of the universal school-based ‘Safe Dates’ curriculum which reduced psychological TDV, moderate physical TDV, sexual TDV perpetration and moderate physical TDV victimization among both boys and girls over a four year follow-up period.

To date, the extant programs demonstrating promise for TDV prevention have been universal programs devised for the general population, while relatively little TDV research has been conducted among high-risk populations (Niolon et al., 2015). Yet, there is growing recognition that youth exposed to violence in the home and/or community are at particularly heightened risk not only for TDV, but multiple forms of subsequent violent delinquency (Crooks et al., 2011; Foshee et al., 2015; Hamby et al., 2012; Baskin and Sommer, 2014; Turner et al., 2016). For example, exposure to intimate partner violence (IPV) during childhood has been linked not only to TDV perpetration (Lichter and McCloskey, 2004) and IPV perpetration as adults (Ehrensaft et al., 2003), but also to general aggression and violence outside of intimate relationships (Brown et al., 2002; Fishbein and Ajzen, 1975; Litrownik et al., 2003). Likewise, exposure to community violence has been linked to perpetration of both violence in the community and TDV (Farrell and Sullivan, 2004; Gorman-Smith et al., 2004; Malik et al., 1997). Given the considerable overlap of risk for disparate types of violence (Wilkins et al., 2014; Klevens et al., 2012; Tolan et al., 1995), it is not surprising that exposure to one form of violence would generalize to the perpetration of multiple forms of violence.

There are likely numerous mechanisms through which exposure to violence confers risk for subsequent perpetration of violence. For instance, witnessing aggression may provide a model for the learning and acceptance of aggressive behaviors by children (Abbey and Jacques-Tiura, 2009). Regardless of the type of exposure – whether witnessing community violence, IPV between parents, or being a victim of physical or sexual abuse – the consequences are similar: exposed youth are more likely to be diagnosed with a psychological disorder and show difficulties with early attachment, emotion regulation, peer relationships, school adjustment, pro-social behaviors, and endorse attitudes condoning violence (Farrell and Sullivan, 2004; Cyr et al., 2010; Guerra et al., 2003; Wekerle and Wolfe, 2003). And, each of these risk factors increases the propensity for perpetrating violence against strangers, peers, and dating partners (Lichter and McCloskey, 2004; Ehrensaft et al., 2003; Herrenkohl et al., 1997; Lansford et al., 2007; Moynihan et al., 2011; Smith and Thornberry, 1995). Thus, there is reason to suspect youth exposed to violence may represent a unique population demonstrating rates of multiple forms of violence, including TDV, that differ from the general population. This heightened risk for multiple forms of violence is an important consideration as these high-risk youth may require the intensity of a selective intervention in place of, or in combination with, universal strategies. As such, identification of targeted cross-cutting violence prevention programs for youth exposed to violence is warranted and necessary (Wilkins et al., 2014).

In its efforts to build the evidence base for programs that prevent males’ sexual violence perpetration, CDC identified the Expect Respect Support Group (ERSG) intervention (Ball et al., 2009, 2012) as a targeted program already in implementation and ready to undergo evaluation. ERSG was initially developed as an empirically-informed TDV prevention program targeting high-risk adolescents with a history of violence exposure. However, the ERSG program addresses a number of shared risk factors for multiple forms of violence (e.g., attitudes condoning violence, emotion regulation, dynamics of health peer relationships) (Ball et al., 2009, 2012), and there is therefore reason to suspect it may impact multiple forms of violence among its high-risk participants.^1^ Notably, an initial pilot study using a single-group design indicated youth demonstrated increases in positive dating behaviors and decreases in emotional and physical TDV from pretest to posttest (Ball et al., 2012). However, this study did not look at other forms of violence. Given these preliminary findings, CDC’s focus on identifying cross-cutting violence prevention strategies (Wilkins et al., 2014), and the current state of implementation of this program, a more rigorous evaluation of the ERSG program’s effects (on multiple forms of violence) is warranted. In the present study, we evaluate the effects of ERSG on TDV, reactive aggression, and proactive aggression. We hypothesized that participation in the ERSG intervention would be associated with significant reductions in all three forms of violence.

## 2. Methods

### 2.1. Participants & procedures

Students from 36 schools in Texas were referred by school counselors or social workers for screening if they suspected, for any reason, the student had been exposed to violence in the home, school, and/or community. During brief intake assessments, semi-structured interviews were conducted to assess if students had ever been the witness, victim, or perpetrator of 1) TDV, 2) IPV between parents, 3) peer violence, 4) child abuse, and/or 5) some other form of violence in the home or community. Youth that verbally endorsed at least one type of violence exposure and were 11–17 years old were eligible to participate in the study. Most students (73%) reported multiple forms of violence exposure. However, this likely an underestimate of violence exposure as we did not exhaustively query each type of violence exposure.^2^

**Fig. 1.**
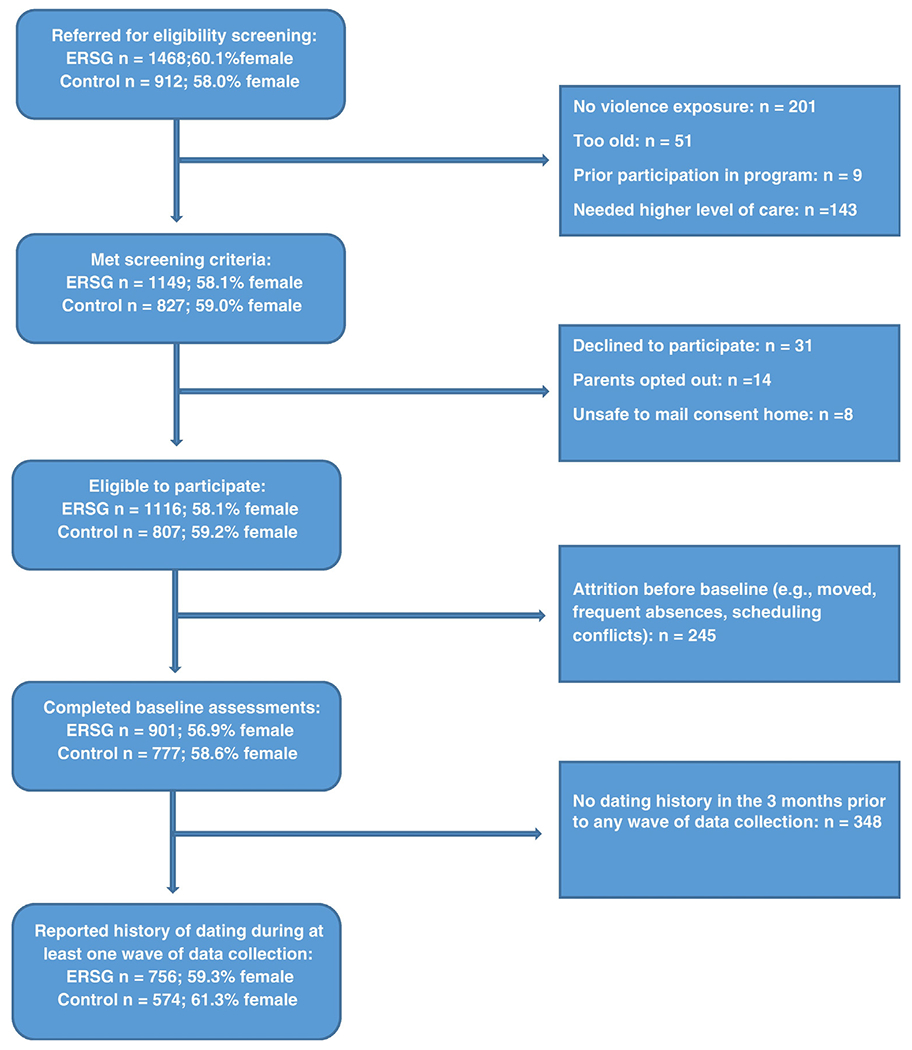
Sample selection procedures.

Students were considered eligible for participation if they 1) endorsed history of violence exposure, 2) had not previously participated in ERSG support groups, 3) were not deemed to need a more significant care, and 4) were between the ages of 11 and 17. Of the 2380 students referred for screening, 1678 (*M*_age_ = 14.3, S.D.= 1.7, Range = 11–17) met inclusion/exclusion criteria, assented, and completed baseline surveys; 1278 students (76.2%) completed surveys at time 2; and 906 students (54.0%) at time 3. Attrition rates were comparable across the treatment conditions (*χ*(1)=0.020, η=0.003; *p*=0.89) and are consistent with longitudinal school-based research (Foshee et al., 2005; Horton and Lipsitz, 2001). There were no significant differences between study completers and non-completers on baseline variables. All students with reactive and proactive aggression data were included in analyses of these outcomes. Only students reporting a history of dating in the preceding three months at one or more waves of data collection were included in the analysis of TDV indices. The analytic dating sample included 1330 students in grades 6–12 (*M_age_*=14.3, *SD*=1.6, Range=11–17). Fig. 1 displays selection procedures for the analytic samples. Overall, the sample was generally representative of the participating school districts in terms of race primarily comprising minority populations. Table 1 provides the breakdown of students by ethnicity and grade level.

ERSG is the core component of a larger comprehensive, multi-level program including school-wide prevention through policy, staff training and education, and youth leadership development provided by the SAFE Alliance: a nonprofit child abuse, domestic violence, and sexual assault victim service provider. This evaluation was focused only on the support group (ERSG) component of the program. The treatment condition included 24 schools already receiving the ERSG intervention. Students in ERSG schools attended up to 25 weekly structured group support sessions focused on developing healthy relationship skills and modifying maladaptive norms about dating behavior.

The curriculum includes five multi-session units: 1) Developing Group Skills (creating group guidelines and practicing communication skills); 2) Choosing Equality and Respect (learning about qualities of healthy relationships, defining abuse and respect, questioning gender stereotypes, and recognizing the use and abuse of power); 3) Recognizing Abusive Relationships (naming the violence, recognizing the impact of violence in your life, and understanding the dynamics and warning signs of dating abuse); 4) Learning Skills for Healthy Relationships (handling jealousy and anger, setting boundaries, asking for consent, resolving conflicts, and ending a relationship); and 5) Getting the Message Out (mixed gender discussion, and standing up against violence). Each group session is structured with a brief check-in (5 min), an educational component (15 min), group activities and discussion (30 min), and a wrap-up (5 min). Group activities, such as educational videos, interactive games, role plays, and creative expression through art and poetry, are designed to engage students in a variety of learning experiences. In keeping with best practices in the use of manualized treatments, support group facilitators work flexibly and creatively with the curriculum and allow time and space for handling crises, individual concerns, and group dynamics. Relationship skills, such as listening, caring, sharing personal experiences, expressing emotions, and problem solving, are practiced as an integral part of the group process. This intervention has been described in great depth elsewhere (Ball et al., 2009, 2012).

All group facilitators attended weekly individual supervision and biweekly group supervision with the counseling manager. Additionally, the counseling manager randomly observed and rated 2 of every facilitator’s sessions for protocol adherence and all facilitators completed fidelity surveys at the end of the first and second semesters. Ninety-five percent of groups focused on the core elements of the ERSG intervention in *all or most of the sessions* and 97% of group facilitators used the provided activities and discussion questions *in all or most of the sessions*.

Groups were conducted separately for boys and girls and facilitated by trained same-sex facilitators to encourage openness among group members and build trust. During the multiple decades of implementation, ERSG has been implemented in various formats including mixed-sex groups and with mixed-sex facilitators. However, based on experience the program creators and implementers believe the same-sex group format is ideal. Feedback from boys in the groups suggests it allows them to “let their guard down,” and feel less pressure to appear tough and strong. Likewise, girls learn they can support each other and be free of relational aggression such as gossip, rumors, and bullying they may have previously experienced. Additionally, same-sex groups seem to achieve a deeper level of self-disclosure for both boys and girls, and at faster rate. The students appear to more easily build trust in the group, understand one another, and empathize with one another.

Twelve “treatment as usual” (TAU) control schools were recruited from two neighboring school districts. School districts were matched by data published by the Texas Education Agency for the 2010–11 school year. Matching data included ethnic composition, economic disadvantage, percent of students with disciplinary placements, and percent of students “at risk” as defined by the Texas Education Agency. TAU services varied by student and school, but generally included individual, short-term, school-based student support and psycho-education intended to help students to identify healthy relationships, develop coping skills, and practice assertive communication. To minimize risk of contamination, separate staff delivered services in the TAU and ERSG treatment conditions.

Participants were recruited in three cohorts over the 2011–2012, 2012–2013, and 2013–2014 school years. Data collection was conducted in 3 waves. Baseline data collection for each cohort occurred during the fall semester (September–January), as close to the start of the school year as possible. Wave 2 of data collection occurred at the end of the school year (April–May) after completion of all ERSG sessions, and the final wave occurred in the first half of the following school year (September–February). See Table 2 for average duration between measurement waves and average age at each wave.

Waivers of parental consent were obtained and informational letters were mailed to parents/guardians allowing them to opt out by mail or phone. During initial interviews, facilitators explained confidentiality and mandatory reporting requirements to students who gave written assent before participating. Students were informed all information would be confidential except for disclosures of child abuse, homicidal, and/or suicidal threat, which were reported to the appropriate agencies specified by law. If students disclosed abuse, group facilitators provided crisis counseling, safety planning, assistance with protective orders, legal advocacy, and referrals as needed. All procedures were approved by CDC’s IRB.

### 2.2. Measures

#### 2.2.1. Dating violence

Students reported on six indices of TDV perpetration and victimization: (**1**) controlling behaviors (5 items,α=0.70& 0.78), (**2**) psychological TDV (8 items, α=0.72 & 0.80), (**3**) physical TDV (5 items, α=0.76 & 0.82), (**4**) sexual TDV (6 items, α=0.69 & 0.76), (**5**) fear/intimidation (3 items, α=0.56 & 0.82), (**6**) injury (3 items, α=0.75 & 79), and one item regarding the use of physical self-defense. Questions were adapted from the Conflict in Adolescent Dating Relationships Inventory (Wolfe et al., 2001) and Safe Dates TDV scales (Foshee et al., 1998). Additional items were developed where necessary to adequately measure constructs. Pilot testing with adapted TDV measures was conducted to confirm reading level, factor structures, and psychometric properties. Students responded to items on a 4-point scale where, 0 = Never, 1 = Rarely, 2 = Sometimes, and 3 = Often. Consistent with prior research on TDV, a 3-month reporting period was utilized due to the short-lived nature of adolescent relationships and to minimize recall error common to retrospective reporting (Orpinas et al., 2013). TDV survey items and instructions are presented in eTable 1 and eMethods 1 of the supplement.

#### 2.2.2. Reactive-proactive aggression

Because many of the skills taught in ERSG may influence multiple forms of violence, we also assessed violence independent of dating relationships. Students completed the Reactive–Proactive Aggression Questionnaire (Raine et al., 2006) comprising 11 reactive aggression items (e.g., “Gotten angry or mad or hit others when teased”; α = 0.85) and 12 proactive aggression items (e.g., “Hurt others to win a game”; α = 0.85). Response options ranged from 0 (Never) to 3 (Often). To be consistent with reporting on TDV and to minimize recall error common to retrospective reporting, a 3-month reporting period was utilized.

## 3. Data analysis

Analyses were conducted separately by sex because the same-sex format of ERSG sessions undoubtedly engendered qualitatively distinct intervention characteristics for boys and girls. For example, focus groups conducted with previous ERSG participants revealed boys reported improved communication skills and anger control, whereas girls reported increased assertiveness, self-esteem, and expectations to be treated with respect by a dating partner (Ball et al., 2009). Additionally, there are pertinent sex differences in the characteristics of dating violence, and previous evaluations of TDV prevention programs have proffered gender specific effects (Reidy et al., 2016; Wolfe et al., 2009).

To assess change in violence outcomes, we estimated linear latent growth curves (LGCs). All analyses were performed with Mplus (version 7.3) controlling for clustering of data within schools (all intra-class correlations ≤ 0.04) with a sandwich estimator. Full information maximum likelihood (FIML) was utilized to handle missing data. FIML is superior to alternative methods of dealing with missing data when data are missing at random (Wang and Wang, 2012). LGCs are particularly ideal for the present data because of their flexibility in handling non-normally distributed repeated measures, partially missing data, and unequally spaced time points (Curran et al., 2010).

Participants in the intervention schools varied substantially in the number of sessions attended, and it was reasonable to assume that the effect of the intervention should be stronger with higher exposure. Therefore, we conducted both intention-to-treat (ITT) analysis (i.e., binary ERSG vs. TAU condition as the predictor of growth curves and intercepts) and dosage analysis (i.e., the number of ERSG sessions attended as the predictor). For the ITT analysis, students in the ERSG condition were coded as 1 regardless of how many, if any, sessions they attended while students in the TAU condition were coded 0. For the dosage analysis, students in the treatment condition received a dosage score of 0 to 25 based on the number of ERSG sessions attended, and students in the TAU control condition were coded as 0 because they attended no ERSG sessions.

The data structure was reorganized such that participants’ age at each measurement wave was used as the metric of time, rather than the wave of assessment. Restructuring the data in this format provides information on the growth trajectory parameters from age 11.0 (the minimum age at Wave 1) to 18.39 (the maximum age at Wave 3) (Reyes et al., 2012). For each dependent variable, the LGC model estimated a latent intercept, which reflects the mean of the violence measure at baseline (age 11), and the average latent mean slope, which reflects the rate of change in the violence outcome (from age 11 to 18). Additionally, both parameters were allowed to vary across individual. To determine the effect of ERSG on changes in violence, the slope parameter for the sample was regressed on the ERSG predictor. The coefficient of this regression reflects the individual’s divergence from the average slope of the sample over time. A negative regression coefficient indicated that, as value of the ERSG predictor increased, the frequency of violence decreased more markedly over time. Therefore, the slope for adolescent ί attending *K* number of ERSG sessions can be computed by adding the fixed slope to the regression coefficient multiplied by number of sessions. With constant slope of −0.05 and a dosage effect of *b* = −0.005, the slope for a student attending 25 sessions would be −0.05 + (−0.005 × 25) = −0.175. We can further calculate an individual’s total change over time by multiplying this slope by time (i.e., age). Thus from age 11 to 18, we would expect an overall decrease of 1.225 in their mean violence score.

## 4. Results

### 4.1. Group characteristics

District level demographic data for the TAU and ERSG districts can be found in eTable 2 of the supplement. Students in treatment schools attended an average of 13.1 ERSG sessions (*SD* = 7.3; range = 0–25; median = 15; mode = 16). Students in TAU schools received an average of 1.1 counseling or psycho-education sessions (*SD*=1.3; range=0–7; median=1; mode=0), with 70.0% receiving 1 session or less.

At baseline, students in TAU were slightly older (*M* = 14.48, *SD* = 1.8) than students in ERSG (*M* = 14.22, *SD* = 1.5); *t*(1326) = 2.83, *p*=0.005, *d* =0.16. There were no differences between the ERSG (*M*=3.1, *SD*=0.9) and TAU (*M*=3.0, *SD*=0.9) on history of dating partners *t*(1275) = 1.44, *p* = 0.15, *d* = 0.09, or biological sex *χ*(1) = 0.58, *p*=0.45 at baseline. Girls in the ERSG condition reported more reactive aggression (*b* = 0.920, *p* = 0.005) and proactive aggression (*b* = 0.535, *p* = 0.001) at baseline than girls in the TAU condition. Boys in the ERSG condition reported more proactive aggression at baseline than boys in the TAU condition (*b*=1.03, *p*=0.001). See Table 3 for means of violence outcomes.

### 4.2. ITT latent growth curves

There were no significant effects for any TDV outcomes. However, youth in the ERSG condition demonstrated declines in reactive (Boys: *b* = −0.030, β = −0.24, *p* = 0.06; Girls: *b* = −0.058, β = −0.53, *p* < 0.001) and proactive (Boys: *b* = −0.061, β = −0.55, *p* < 0.001; Girls: *b* = −0.032, β = −0.36, *p* < 0.001) aggression.

### 4.3. Dosage latent growth curves

Average intercepts and slopes of all outcomes when ERSG dosage is 0 are presented in eTable 3. There were incremental reductions in reactive and proactive aggression, psychological and sexual perpetration, and psychological, physical, and sexual victimization for boys attending ERSG sessions as indicated by the significant negative slope (Table 4). Additionally, the intercepts for each of these outcomes were significantly positively associated with dosage, indicating that those boys who had higher initial mean scores on these outcomes attended more ERSG sessions.

Among girls, ERSG dosage was associated with significant decreases for reactive and proactive aggression (Table 4). Additionally, there was a significant effect of the intercept regressed on ERSG dosage, suggesting that girls with higher initial reactive aggression scores attended more ERSG sessions. There was a marginal effect suggesting potential declines in physical TDV perpetration. There was also a converse marginal effect for sexual TDV victimization among girls wherein attending more ERSG sessions was associated with an increase in victimization (Table 4).

## 5. Discussion

We examined the effectiveness of the ERSG program in reducing TDV and general aggression among high-risk adolescents (i.e., youth exposed to violence) over the course of seven years in an accelerated research design. Findings indicate the number of ERSG sessions attended related to incremental declines for multiple TDV and aggression outcomes, particularly for boys. We found no effects for boys or girls on reported self-defense, controlling behaviors, fear/intimidation, or injury. For girls, ERSG dosage was associated with decreases in reactive and proactive aggression. There was also a marginal effect suggesting a possible increase in sexual TDV victimization among girls attending ERSG. It is unclear how or why ERSG might contribute to an increase in sexual victimization. It is possible that this trend is merely statistical artifact considering it was identified only for this outcome and it was not significant at the α=0.05 level. Alternatively, there was an additional trend indicating that the girls reporting less sexual victimization at baseline completed more sessions, and conversely, girls reporting more sexual victimization at baseline completed the fewest sessions. Given the ERSG focus on educating youth about sexual TDV behaviors, it is possible that these trends together reflect an increased awareness of sexual TDV victimization among girls that had reported less sexual victimization at baseline, rather than an increase in actual victimization. This increased awareness would, in turn, translate into a consequent increase in reporting. Regardless, this potential effect of the ERSG program on girls’ sexual victimization warrants continued investigation. Any increase in victimization may arguably negate benefits related to reductions in perpetration.

The ERSG program does appear to be a promising strategy to reduce TDV and general violence among boys that have been exposed to violence. ERSG was associated with reductions in reactive and proactive aggression, psychological and sexual TDV perpetration, and psychological, physical, and sexual TDV victimization. Importantly, we found that ERSG dosage (i.e., number of sessions attended) was significantly associated with initial levels of violence among boys. That is, boys reporting the most violence perpetration and victimization at baseline, attended the most ERSG sessions. Thus, the highest-risk boys (i.e., most violent at baseline) attended the most sessions and demonstrated the most reduction in violence therefore benefitting most from this program. This is counter to what we might expect based on selective attrition bias common to intervention research, wherein, treatment completers generally have a better preexisting prognosis than non-completers (Larzelere et al., 2004). Hence, we would expect the highest-risk youth to drop out first. Yet, in our sample it was the less violent boys who attended fewer sessions while the high-risk boys attended the most sessions and demonstrated more behavior change over time. This finding underscores the potential utility of the ERSG program for high-risk boys both in sustaining their engagement and impacting change. Moreover, standardized effects (β’s ranging from −0.20 to −0.56) were moderate to large in size (Cohen, 1988) further indicating substantial impact of ERSG for these high-risk boys.

### 5.1. Limitations

Despite attempts to maximize rigor and control for potential confounds in the present design, only a true randomized experimental design allows us to draw causal inferences. Yet, randomized controlled trials are often impractical and potentially unethical in situations such as this where an intervention is already in place (Glass et al., 2013). In this context, it is incumbent upon us as public health researchers to utilize the best alternative designs to maximize our ability to support causal claims (Glass et al., 2013). Additionally, we lost a large percentage of our sample to attrition; a common issue in longitudinal school-based research and consistent with prior clinical trials (Foshee et al., 2005; Horton and Lipsitz, 2001). This is a critical issue because the highest-risk students are often absent from school and move frequently and therefore least likely to be retained. Of course, the highest-risk boys in our sample were the most likely to persist in the intervention potentially mitigating this weakness. Furthermore, because TDV is dyad dependent, reports by dating partners of the respondents may provide more accurate data on TDV perpetration and victimization in future research.

Finally, it is important to note that although the present evaluation proffered moderately large effect sizes, the reductions in violence were fractional. This signals the need to develop comprehensive, multi-pronged prevention approaches to most effectively curb the evolution of violence among these high-risk youth. Evaluations of TDV programs like ‘Shifting Boundaries’ (Taylor et al., 2015) underscore the importance of environmental strategies to prevent violence. Merging such universal strategies with the ERSG program may bolster the prevention effects. In fact, future research should evaluate ERSG in the context of the program’s other universal strategies which were not the focus of this study (school-wide prevention including school policy, staff training, education and youth leadership development to see whether this multi-pronged prevention effort magnifies the effects of ERSG. In a related vein, we did not collect school level data in the present evaluation. Future research would be strengthened by examining how school factors (e.g., resources, graduation rates, enrollment size, staff-student ratios, presence of resource officers, community crime rates, etc.) may contribute to violence and affect response to the ERSG intervention. In spite of these limitations, the present findings merit optimism and compel further evaluation of ERSG and pertinent factors that may influence its effectiveness.

### 5.2. Public health implications

Exposure to violence in the home, school, and/or community increases the risk for multiple forms of violence among youth (Crooks et al., 2011; Foshee et al., 2015; Hamby et al., 2012; Baskin and Sommer, 2014; Turner et al., 2016; Lichter and McCloskey, 2004; Ehrensaft et al., 2003; Brown et al., 2002; Fishbein and Ajzen, 1975; Litrownik et al., 2003; Farrell and Sullivan, 2004; Gorman-Smith et al., 2004; Malik et al., 1997). There are a myriad of chronic behavioral, social, mental and physical health consequences for victims and perpetrators of such violence (Kann et al., 2014; Banyard and Cross, 2008; Ackard et al., 2007; Smith et al., 2003; Vagi et al., 2015; Campbell, 2002; McLaughlin et al., 2016). Moreover, youth who are victims of TDV are more likely to be in future violent relationships as adults (White and Smith, 2004) compounding violence sequelae from adolescence with the violence sequelae in adulthood (Campbell, 2002). Thus, it seems that the ERSG program has cross-cutting potential to prevent a myriad of adverse health outcomes.

There are also significant economic consequences for the victims, perpetrators, and society as whole. These costs are important to consider when evaluating the feasibility of implementing prevention programs, especially targeted programs with high resource costs. For instance, the cost to implement two groups (i.e., 25 meetings generally serving 20 youth – 10 boys and 10 girls – over the 9-month academic year) of the ERSG program is approximately $13,000 – primarily salary support for group facilitators.^3^ This may be an unfeasible cost to expect schools to absorb, especially considering the schools most in need of prevention programs like ERSG often have the fewest resources and many students in need. Yet, the ERSG implementation costs are considerably less than most existing efficacious youth violence programs (Fagan and Catalano, 2013); and, the per-individual cost of the ERSG program (i.e., $650) compared to the per-incident cost of violence argues the necessity of implementing such prevention strategies. For instance, in the U.S., the average cost of a violent assault treated in an emergency department is approximately $7052; approximately $162,755 for assaults requiring hospitalization; and more than $1.5 million in health-care and lost wages for each assault resulting in death (Centers for Disease Control and Prevention, n.d.).^4^ Cohen and Piquero (2009) have estimated that intervening at birth with high-risk youth would save between $2.6 and $4.4 million per individual by the time they reached adolescence. In other words, the expense to implement intensive prevention programs such as ERSG demonstrates significant returns on investment in terms of future savings (Fagan and Catalano, 2013). As such, it seems that it may be worthwhile for prevention researchers, practitioners, and policy makers to collaborate in innovation of economic strategies to fund the implementation of these resource-intensive programs.

In the United States, youth represent 35% of all homicide victims and approximately 50% of all homicide perpetrators (Cooper and Smith, 2011). Male youth are disproportionately represented both as victims and perpetrators in these statistics while females are more likely to be killed by their male intimate partners and to be the victims of sex-related homicides by males (Cooper and Smith, 2011). Thus, results of the present evaluation indicating ERSG’s effectiveness with the most violent boys for both youth violence and sexual violence perpetration suggest this program may be most likely to have an impact on the population with the greatest rates of violence.

In sum, targeted prevention programs such as ERSG that affect multiple types of violence proffer the opportunity prevent life-course trajectories of chronic health problems and hundreds of millions in economic burden on society (Cohen and Piquero, 2009). Our findings, while requiring replication, suggest ERSG could be an effective strategy to prevent psychological and sexual TDV perpetration as well as reactive and proactive aggression among boys exposed to violence. In particular, this intervention may be optimal for engaging the most violent of these boys therefore translating into the greatest health benefits and economic savings.

## Supplementary Material

Appendix A. Supplementary data

## Figures and Tables

**Table 1:** Demographic information.

	Boys				Girls			
	TAU		ERSG		TAU		ERSG	
	*N*	%	*N*	%	*N*	%	*N*	%
Race/ethnicity								
Hispanic/Latino	133	41.3	195	50.3	208	45.7	316	61.6
African-American	82	25.5	73	18.8	78	17.1	58	11.3
White	34	10.6	56	14.4	67	14.7	60	11.7
Multi-racial	51	15.8	45	11.6	75	16.5	56	10.9
Other	15	4.6	15	3.9	23	5.1	18	3.6
DNR	7	2.2	4	1.0	4	0.9	5	1.0
Grade level								
Grade 6	50	15.5	3	0.8	32	7.0	0	0
Grade 7	60	18.6	50	12.9	91	20.0	87	17.0
Grade 8	60	18.6	144	37.1	86	18.9	176	34.3
Grade 9	59	18.3	57	14.7	60	13.2	62	12.1
Grade 10	34	10.6	64	16.5	61	13.4	80	15.6
Grade 11	25	7.8	51	13.1	68	14.9	75	14.6
Grade 12	28	8.7	14	3.6	52	11.4	29	5.7
DNR	6	1.9	5	1.3	5	1.1	4	0.8

Note. TAU=Treatment as Usual; ERSG=Expect Respect Support Groups; DNR=Did not respond.

**Table 2:** The duration (in months) of data collection waves and age (in years) at each wave.

Duration	Wave 1 → Wave 2				Wave 2 → Wave 3				Wave 1 → Wave 3			
	Min	Max	M	SD	Min	Max	M	SD	Min	Max	M	SD

Boys	2.27	8.84	5.79	1.23	3.29	10.35	5.92	1.64	6.67	16.85	11.79	2.04
Girls	2.04	8.51	5.77	1.26	3.29	10.41	6.10	1.60	6.77	16.95	11.89	1.96
Age	Age at Wave 1				Age at Wave 2				Age at Wave 3			
	Min	Max	M	SD	Min	Max	M	SD	Min	Max	M	SD

Boys	11.0	17.0	14.15	1.59	11.25	17.74	14.50	1.57	11.64	18.31	14.76	1.46
Girls	11.0	17.0	14.34	1.69	11.22	17.67	14.67	1.67	11.62	18.39	14.92	1.52

Note. Min = Minimum; Max= Maximum; M = Mean; SD= Standard Deviation.

**Table 3:** Means and variances for all outcome variables by condition.

Outcome variables	TAU						ERSG					
	Wave 1		Wave 2		Wave 3		Wave 1		Wave 2		Wave 3	
	M	Var	M	Var	M	Var	M	Var	M	Var	M	Var
Boys												
Self-defense	0.339	0.69	0.267	0.55	0.278	0.65	0.380	0.80	0.312	0.60	0.203	0.37
Controlling perpetration	0.481	0.27	0.363	0.17	0.327	0.21	0.525	0.32	0.442	0.28	0.374	0.24
Psychological perpetration	0.143	0.10	0.103	0.05	0.080	0.03	0.192	0.12	0.181	0.11	0.148	0.09
Physical perpetration	0.043	0.04	0.033	0.04	0.013	0.01	0.088	0.08	0.095	0.09	0.058	0.08
Sexual perpetration	0.097	0.07	0.058	0.04	0.054	0.01	0.161	0.10	0.139	0.09	0.088	0.08
Fear perpetration	0.112	0.11	0.066	0.05	0.067	0.05	0.104	0.11	0.099	0.12	0.097	0.10
Injury perpetration	0.114	0.20	0.077	0.12	0.104	0.13	0.105	0.12	0.098	0.10	0.077	0.09
Controlling victimization	0.661	0.41	0.532	0.34	0.487	0.37	0.677	0.38	0.558	0.35	0.478	0.33
Psychological victimization	0.215	0.14	0.149	0.08	0.105	0.08	0.249	0.14	0.240	0.16	0.182	0.12
Physical victimization	0.128	0.15	0.098	0.09	0.047	0.03	0.162	0.14	0.182	0.22	0.081	0.10
Sexual victimization	0.157	0.09	0.094	0.05	0.100	0.05	0.252	0.16	0.195	0.11	0.146	0.09
Fear victimization	0.070	0.07	0.066	0.06	0.059	0.05	0.085	0.08	0.073	0.09	0.064	0.09
Injury victimization	0.172	0.26	0.109	0.19	0.167	0.23	0.164	0.21	0.151	0.20	0.116	0.13
Reactive aggression	1.199	0.38	1.066	0.40	0.950	0.34	1.259	0.37	1.099	0.40	0.952	0.37
Proactive aggression	0.336	0.15	0.293	0.15	0.229	0.12	0.487	0.26	0.430	0.22	0.307	0.15
Girls												
Self-defense	0.290	0.56	0.225	0.45	0.159	0.33	0.368	0.66	0.296	0.54	0.215	0.33
Controlling perpetration	0.493	0.26	0.393	0.21	0.313	0.18	0.475	0.27	0.365	0.20	0.349	0.21
Psychological perpetration	0.231	0.10	0.193	0.09	0.184	0.08	0.276	0.15	0.196	0.07	0.194	0.07
Physical perpetration	0.079	0.06	0.064	0.05	0.064	0.04	0.149	0.11	0.101	0.06	0.064	0.03
Sexual perpetration	0.095	0.04	0.071	0.02	0.062	0.02	0.130	0.06	0.110	0.05	0.085	0.03
Fear perpetration	0.073	0.06	0.046	0.05	0.038	0.02	0.094	0.09	0.064	0.06	0.021	0.01
Injury perpetration	0.058	0.07	0.041	0.03	0.030	0.02	0.101	0.09	0.063	0.06	0.044	0.04
Controlling victimization	0.718	0.52	0.545	0.33	0.534	0.38	0.672	0.47	0.535	0.34	0.550	0.41
Psychological victimization	0.314	0.20	0.250	0.19	0.234	0.13	0.306	0.19	0.233	0.10	0.241	0.15
Physical victimization	0.082	0.10	0.076	0.07	0.054	0.04	0.136	0.14	0.080	0.05	0.077	0.08
Sexual victimization	0.196	0.14	0.146	0.10	0.122	0.07	0.201	0.13	0.160	0.11	0.142	0.11
Fear victimization	0.217	0.29	0.156	0.23	0.122	0.17	0.203	0.27	0.118	0.13	0.101	0.14
Injury victimization	0.109	0.15	0.078	0.08	0.062	0.04	0.154	0.15	0.105	0.12	0.093	0.09
Reactive aggression	1.249	0.39	1.121	0.42	0.075	0.33	1.344	0.43	1.243	0.43	1.083	0.43
Proactive aggression	0.266	0.11	0.230	0.11	0.201	0.08	0.358	0.17	0.312	0.16	0.226	0.13

Note. TAU = Treatment as Usual; ERSG = Expect Respect Support Groups; M = Mean; Var = Variance.

**Table 4:** Parameter estimates for the regression of latent sample slopes and intercepts on ERSG dosage.

Outcome variables	Intercept boys			Slope boys			Intercept girls			Slope girls		
	*b*	β	*p* ^b^	*b*	β	*p* ^a^	*b*	β	*p* ^b^	*b*	β	*p* ^a^
Self-defense	0.021	0.17	0.53	−0.001	−0.07	0.55	−0.015	−0.08	0.94	0.001	0.10	0.93
Controlling perpetration	0.012	0.11	0.57	−0.001	−0.14	0.60	−0.008	−0.09	0.63	0.000	0.06	0.70
Psychological perpetration	**0.026**	**0.47**	**0.05**	**−0.002**	**−0.56**	**0.03**	0.003	0.08	0.78	0.000	−0.10	0.79
Physical perpetration	0.051	0.22	0.70	−0.001	−0.22	0.71	0.010	0.55	0.18	**−0.001**	**−0.29**	**0.08**
Sexual perpetration	**0.023**	**0.28**	**0.06**	**−0.001**	**−0.20**	**0.04**	0.000	0.02	0.95	0.000	0.00	0.80
Fear perpetration	0.009	0.10	0.45	0.000	−0.08	0.52	−0.003	−0.06	0.56	0.000	0.10	0.94
Injury perpetration	0.002	0.02	0.86	0.000	−0.08	0.90	0.008	0.12	0.22	0.000	0.10	0.20
Controlling victimization	0.026	0.19	0.24	−0.002	−0.21	0.22	−0.029	−0.36	0.14	0.002	0.31	0.16
Psychological victimization	**0.027**	**0.28**	**0.03**	**−0.002**	**−0.28**	**0.02**	−0.011	−0.21	0.53	0.001	0.19	0.38
Physical victimization	**0.036**	**0.43**	**0.01**	**−0.002**	**−0.56**	**0.01**	−0.003	−0.04	0.95	0.000	0.05	0.94
Sexual victimization	**0.028**	**0.26**	**0.06**	**−0.002**	**−0.32**	**0.04**	**−0.017**	**−0.78**	**0.08**	**0.001**	**0.28**	**0.07**
Fear victimization	0.018	0.19	0.39	−0.001	−0.19	0.15	−0.009	−0.17	0.47	0.001	0.16	0.46
Injury victimization	0.001	0.01	0.95	0.000	−0.05	0.97	−0.008	−0.41	0.34	0.001	0.28	0.34
Reactive aggression	**0.037**	**0.35**	**0.02**	**−0.003**	**−0.47**	**0.01**	**0.042**	**0.32**	**0.01**	**−0.003**	**−0.37**	**0.01**
Proactive aggression	**0.049**	**0.39**	**0.001**	**−0.003**	**−0.47**	**0.001**	0.017	0.15	0.10	**−0.001**	**−0.15**	**0.05**

Note. *b* = Unstandardized regression coefficient; β = Standardized regression coefficient; *p* = significance level.

Values in bold print are significant.

a One tailed.

b Two tailed.

## Footnotes

1 To obtain a copy of the Expect Respect Program Manual, which includes the support group curriculum, contact bball@safeaustin.org.

2 ERSG intake interviews are conducted with several goals in mind: 1) screen students for violence exposure and determine eligibility for the program; 2) begin building a relationship with the students and offer a safe space where disclosures can occur; and 3) invite the students to participate in the program and obtain their assent for participation. Staff conducting the intake are trained to balance the tasks of building rapport and assessing if students have been the witness, victim or perpetrator of teen dating violence, IPV between parents, peer violence, child abuse and/or other form of violence in the home or community. Interviews are semi-structured, which means the ERSG staff do not simply read a list of screening questions to the students, but are asking these questions as they engage the students in sharing about their history, living environment, strengths, and experiences with violence. ERSG staff work to empower students and take care not to push for disclosures when students are not ready to work through the consequences, or may refrain from asking more questions about other forms of violence when they notice a student shutting down. Typically, students will disclose additional violence exposures during the course of the group sessions. However, the overwhelming majority of students report multiple forms of violence exposure in this first interview.

3 Intake and additional individual school-based sessions (advocacy/counseling) for group and non-group members as needed are also included in these cost estimates.

4 These dollar amounts are updated to 2015 dollars from 2010 dollars using the Consumer Price Index.
