# Supplementary material for: Evaluation of the expect respect support group program: A violence prevention strategy for youth exposed to violence
Source: Prev Med. Author manuscript; Available in PMC 2025 Feb 5. (PMC11798122; doi:10.1016/j.ypmed.2017.05.003)
Supplement: Appendix A. Supplementary data [file NIHMS950320-supplement-Appendix_A__Supplementary_data.docx]

**SUPPLEMENTAL MATERIALS**

1. **eMethod 1**: Instructions for Dating Violence Survey Measures
2. **eTable 1**: Dating Violence Survey Items
3. **eTable 2**. Demographic Composition of Treatment and Control School Districts during the 2010
   2011 School Year Based on Data Published by the Texas Education Agency.
4. **eTable 3**. Unstandardized Latent Sample Intercepts and Slopes when ERSG Dosage Equals Zero

**eMethods 1. Instructions for** **Dating Violence Survey Measures**
 Students reported on six indices of TDV perpetration and victimization. Question were asked twice, once in relation to the participant’s behaviors toward a dating partner, and once in relation to a partner’s behavior toward the participant. Responses to each question for the six perpetration and six victimization scales were summed to create an index of each type of TDV: Controlling (5 items, *α* = .70 & .78), Psychological (8 items, *α* = .72 & .80), Physical (5 items, *α* = .76 & .82), Sexual (6 items, *α* = .69 & .76), Fear/intimidation (2 & 3 items, *α* = .56 & .82) and Injury (3 items, *α* = .75 & 79). In addition, students responded to one item assessing their use self-defense in dating relationships. Instructions to the dating violence items read,
 *“When you answer the following questions, please think about all the dating relationship(s) you’ve had in the past 3 months. By dating partner we’re talking about a boyfriend or girlfriend, someone you go out with or hang out with in a romantic way, or someone you hook up with. This can be a relationship of any length. In the past 3 months, how often did the following things happen in your dating relationship(s)?”* Participants responded on a 4-point Likert-type scale where, 0 = Never, 1 = Rarely, 2 = Sometimes, and 3 = Often. Consistent with prior research on TDV, a 3-month reporting period was chosen due to the short-lived nature of adolescent relationships as well as to minimize recall error common to retrospective reporting.

**eTable 1.** Dating Violence Survey Items.

| **Perpetration** | | **Victimization** | |
| --- | --- | --- | --- |
| **Controlling Behaviors** | | | |
| I tried to keep my partner from spending time with other people. |  | | My partner tried to keep me from spending time with other people. |
| I checked where my partner was and who he/she was hanging out with. |  | | My partner checked where I was and who I was hanging out with. |
| I did not let my partner do things with other people. |  | | My partner did not let me do things with other people. |
| I showed up at my partner's home, school or work or waited for him/her even when my partner didn't want me to. |  | | My partner showed up at my home, school or work or waited for me even when I didn't want him/her to. |
| I repeatedly used cell phone, text messaging, or e-mail to check up on my partner and to see where he/she was. |  | | My partner repeatedly used cell phone, text messaging, or e-mail to check up on me and to see where I was. |
|  | | | |
| **Psychological TDV** | | | |
| I put my partner down. |  | | My partner put me down. |
| I made fun of my partner in front of others. |  | | My partner made fun of me in front of others. |
| I threatened to end the relationship if my partner didn't do what I wanted. |  | | My partner threatened to end the relationship if I didn't do what he/she wanted. |
| I threatened to hurt or hit my partner. |  | | My partner threatened to hurt or hit me. |
| I yelled and screamed at my partner. |  | | My partner yelled and screamed at me. |
| I made nasty comments about my partner to others. |  | | My partner made nasty comments about me to others. |
| I used e-mails, text messaging, web chat, blog Facebook, MySpace, or Twitter to spread rumors about my partner. |  | | My partner used e-mails, text messaging, web chat, blog Facebook, MySpace, or Twitter to spread rumors about me. |
| I called my partner bitch, slut, ho, player, or gay. |  | | My partner called me bitch, slut, ho, player, or gay. |
|  | | | |
| **Physical TDV** | | | |
| I scratched or slapped my partner. |  | | My partner scratched or slapped me. |
| I hit my partner with a fist or a hard object. |  | | My partner hit me with a fist or a hard object. |
| I twisted my partner's arm or bent his/her fingers. |  | | My partner twisted my arm or bent my fingers. |
| I pushed, shoved, or kicked my partner. |  | | My partner pushed, shoved, or kicked me. |
| I beat my partner up. |  | | My partner beat me up. |
|  | | | |

| **Sexual TDV** | | |  |
| --- | --- | --- | --- |
| I showed nude or almost nude pictures/video of my partner to others. |  | My partner showed nude or almost nude pictures/video of me to others. |  |
| I told my partner that he/she should touch me in a sexual way to prove their love. |  | My partner told me that I should touch him/her in a sexual way to prove my love. |  |
| I spread sexual rumors about my partner. |  | My partner spread sexual rumors about me. |  |
| I pressured my partner to engage in a sexual act. |  | My partner pressured me to engage in a sexual act. |  |
| I grabbed or touched my partner's private parts without his/her consent. |  | My partner grabbed or touched my private parts without my consent. |  |
| I forced my partner to kiss me. |  | My partner forced me to kiss him/her. |  |
| I got my partner drunk or high to get him/her to do something sexual. |  | My partner got me drunk or high to get me to do something sexual. |  |
|  | | |  |
| **Fear/Intimidation** | | |  |
| My partner was afraid of me. |  | I was afraid of my partner. | |
| My partner felt unsafe. |  | I felt unsafe. | |
|  |  | I worried that I could get hurt physically. | |
|  | | |  |
| **Injury** | | |  |
| My partner had a bruise or small cut. |  | I had a bruise or small cut. |  |
| My partner felt physical pain that still hurt the next day. |  | I felt physical pain that still hurt the next day. |  |
| My partner went to a doctor or nurse because of an injury. |  | I went to a doctor or nurse because of an injury. |  |
|  |  |  |  |
| **Self-Defense** | | |  |
| I used physical force to protect or defend myself. |  |  |  |
|  |  |  |  |

**eTable 1.** Dating Violence Survey Items continued.

| **Perpetration** | **Victimization** |
| --- | --- |

| **Demographic Characteristics** | **TAU** | **ERSG** |
| --- | --- | --- |
|  |  |  |
| African-American | 22.2 | 9.5 |
| Hispanic | 53.3 | 60.3 |
| Caucasian | 17.1 | 24.3 |
| American-Indian | 0.4 | 0.3 |
| Asian | 4.6 | 3.3 |
| Pacific Islander | 0.2 | 0.1 |
| 2 or More Races | 2.3 | 2.2 |
| Economically Disadvantaged | 67.9 | 64.0 |
| Limited English Proficiency | 26.0 | 28.7 |
| Students with Disciplinary Placements | 2.0 | 2.0 |
| Students "At Risk" | 55.8 | 48.4 |

**eTable 2.** Demographic Composition of Treatment and Control School Districts during the 2010-2011 School Year Based on Data Published by the Texas Education Agency.

**Note.** Values are percentages.

**eTable 3.** Unstandardized Latent Sample Intercepts and Slopes when ERSG Dosage Equals Zero.

| **Outcome Variables** | **Boys** | | | |  | | **Girls** | | | | |
| --- | --- | --- | --- | --- | --- | --- | --- | --- | --- | --- | --- |
|  | **Intercept** | ***p*** | **Slope** | ***p*** | |  | | **Intercept** | ***p*** | **Slope** | ***p*** |
|  |  |  |  |  | |  | |  |  |  |  |
| Self-Defense | 1.179 | .02 | -.061 | .06 | |  | | .950 | .09 | -.048 | .17 |
|  |  |  |  |  | |  | |  |  |  |  |
| Controlling Perpetration | .294 | .17 | .008 | .62 | |  | | .454 | .03 | -.002 | .86 |
| Psychological Perpetration | -.198 | .10 | .021 | .01 | |  | | .067 | .62 | .010 | .30 |
| Physical Perpetration | .077 | .63 | -.001 | .91 | |  | | .047 | .86 | .002 | .89 |
| Sexual Perpetration | -.044 | .65 | .008 | .23 | |  | | .166 | .01 | -.006 | .16 |
| Fear Perpetration | .302 | .001 | -.015 | .01 | |  | | .139 | .01 | -.006 | .04 |
| Injury Perpetration | .405 | .001 | -.022 | .001 | |  | | .129 | .13 | -.005 | .33 |
|  |  |  |  |  | |  | |  |  |  |  |
| Controlling Victimization | .301 | .29 | .019 | .36 | |  | | .607 | .02 | .001 | .95 |
| Psychological Victimization | -.340 | .05 | .036 | .01 | |  | | .054 | .77 | .015 | .27 |
| Physical Victimization | .067 | .82 | .001 | .79 | |  | | -.077 | .78 | .011 | .52 |
| Sexual Victimization | .146 | .36 | -.001 | .91 | |  | | .262 | .09 | -.007 | .50 |
| Fear Victimization | .121 | .25 | -.004 | .53 | |  | | .116 | .43 | .003 | .74 |
| Injury Victimization | .624 | .001 | -.034 | .01 | |  | | .168 | .42 | -.004 | .74 |
|  |  |  |  |  | |  | |  |  |  |  |
| Reactive Aggression | 2.063 | .001 | -.064 | .001 | |  | | 2.501 | .001 | -.089 | .001 |
| Proactive Aggression | .881 | .001 | -.038 | .001 | |  | | .952 | .001 | -.047 | .001 |
|  |  |  |  |  | |  | |  |  |  |  |

**Note.** *p* = signficance level
